# Development and evaluation of scenario-based e-simulation for humanitarian health training: a mixed-methods action research study

**DOI:** 10.1136/bmjopen-2023-079681

**Published:** 2024-08-05

**Authors:** Awsan Abdullah Saeed Bahattab, Omar Zain, Monica Linty, Nieves Amat Camacho, Johan Von Schreeb, Ives Hubloue, Francesco Della Corte, Luca Ragazzoni

**Affiliations:** 1 CRIMEDIM – Center for Research and Training in Disaster Medicine, Humanitarian Aid and Global Health, Università del Piemonte Orientale, Novara, Italy; 2 Department for Sustainable Development and Ecological Transition, Università del Piemonte Orientale, Vercelli, Italy; 3 Department of Community Medicine and Public Health, University of Aden, Aden, Yemen; 4 Department of Global Public Health, Center for Research on Health Care in Disasters, Karolinska Institutet, Stockholm, Sweden; 5 ReGEDiM – Research Group on Emergency and Disaster Medicine, Vrije Universiteit Brussel, Brussel, Belgium; 6 Department of Translational Medicine, University of Eastern Piedmont, Novara, Italy

**Keywords:** PUBLIC HEALTH, MEDICAL EDUCATION & TRAINING, Internet, QUALITATIVE RESEARCH, Surveys and Questionnaires, eHealth

## Abstract

**Abstract:**

**Objectives:**

This study aimed to develop and evaluate a scenario-based e-simulation (SBES) to address the limited avilability of accessible and practical training for humanitarian public health responders. The objectives included SBES customisation, effectiveness evaluation, and identifying learning-enhancing design elements.

**Design:**

A university-based, mixed-methods action research design.

**Setting:**

The study was conducted at an international university’s academic centre in Italy, and at a university-based master’s programme in Yemen.

**Participants:**

The study involved 20 multidisciplinary global health and education experts and 66 international medical and health sciences students.

**Results:**

Between September 2020 and July 2022, four SBES modules were developed, implemented and evaluated using a rapid prototype model. The modules, which targeted health professionals new to or with limited experience in the humanitarian field, included health needs assessment, essential health services, communicable diseases and health system. Formative evaluation improved the design and implementation of the SBES, which was found to be effective in the summative evaluation, evident from positive student reactions (the overall mean satisfaction rate was 6.03 out of 7, 95% CI 5.95 to 6.47) and the significant improvement in knowledge scores (p<0.001, effect size: 1.179). The identified effective design of SBES includes overlapping elements among content, strategy and technology. Poor internet access was recognised as a potential barrier to delivering the training in the humanitarian context, highlighting the need to develop an offline version in the next phase.

**Conclusion:**

The developed SBES met the training needs of the academic institution involved. The study findings will contribute to advancing future SBES training initiatives for disaster medicine and global health. Further studies are recommended to evaluate and address the challenges associated with SBES implementation beyond the study setting.

**STRENGTHS AND LIMITATIONS OF THIS STUDY**

- Mixed-methods action research enabled comprehensive and generalisable training needs analysis and allowed the generation of valuable insights into the evaluation of scenario-based e-simulations (SBES), including a theoretical understanding of effective design elements and effectiveness of the training outcomes.
- The iterative and cyclical nature of action research facilitated practical adjustments in designing and implementing SBES.
- The involvement of diverse stakeholders brought multiple perspectives that enriched the study’s findings.
- While the subjective nature of action research and the limited scope of outcome evaluation restrict generalisability beyond the study setting, action research remains valuable for providing practical and effective solutions in real-world contexts.

## Introduction

 Responding to the surge in humanitarian crises, the humanitarian sector has grown significantly over the past few decades, with health professionals forming a substantial part of its workforce.^1^ These professionals must not only save lives but also address broader public health issues.^2^ However, health professionals often lack sufficient public health training.^3^ There is a need for humanitarian health professionalisation through standardised education and training programmes. However, several challenges exist, including the lack of a comprehensive competency framework, and limited availability and accessibility of these programmes.^4^ Furthermore, existing education and training programmes tend to be theory-based,^4^ and practical field trainings that allow trainees to apply their knowledge in a humanitarian context are lacking.^3^

While simulation is increasingly recognised as an effective teaching method for global health education,^5^,^14^ the logistical challenges associated with developing on-site simulations pose a significant obstacle.^3^ If properly developed, innovative e-learning solutions such as e-simulation can address the limitations of theoretical teaching methods by bridging the knowledge-application gap and enhancing accessibility.^3 15^ Moreover, the scalability of e-simulation can reduce long-term training costs by reaching more trainees over time.^16^ Despite the growing trend of simulation as a teaching method in humanitarian health education,^17^,^26^ the use of e-simulation remains rare,^7 27^ and educational theories are rarely integrated into its design.^28^ The lack of a theoretical foundation for e-simulation challenges the notion of simulation effectiveness.

Scenario-based e-simulation (SBES) is a conceptual simulation well suited for teaching public health skills, and encompassing cognitive, metacognitive and affective skills.^29^,^31^ However, there is a lack of literature on the utilisation, design and evaluation of SBES, which presents a significant obstacle for educators seeking to bridge the gaps in teaching and training methods within humanitarian health education.

Parallel with the global needs, there was a need for innovative humanitarian public health training in the academic training centre where the study was conducted. To address these gaps, this study aimed to develop and evaluate an SBES to meet the demands for public health in humanitarian crises training. The specific objectives of the evaluation were to tailor the design and implementation of the SBES to meet the targeted trainees' needs, assess its effectiveness and identify the design elements that enable learning transfer. Hence, this paper aims to present the results of the analysis, design, development and evaluation phases of iterative action cycles.

## Methods

To allow for transparent and detailed reporting, the methods and results sections were guided by the Criteria for Describing and Evaluating Training Interventions in Healthcare Professions Checklist.^32^

### Study design

The study design consists of a nested mixed-methods action research approach, where quantitative research components (quasi-experiment, survey) are embedded within qualitative research.^33^,^35^ While the qualitative methods aimed to explore the SBES design elements perceived by the trainees as effective for knowledge transfer and the action cycles customised the gaps in design, the quantitative methods aimed to measure the outcomes of SBES training in terms of knowledge and satisfaction.

### Setting

CRIMEDIMThe study was conducted at CRIMEDIM (Centre for Research and Training in Disaster Medicine, Humanitarian Aid, and Global Health) - Università del Piemonte Orientale (Novara, Italy), between September 2020 and July 2022. Additionally, the SBES was piloted within the Master of Public Health programme at the University of Aden (Yemen).University of Aden (Yemen).

### SBES target population and study participants

The SBES targeted health professionals responding to public health crisis in humanitarian settings. A total of 66 students and 20 experts participated in the study. For qualitative evaluation, participants were purposively sampled, including experts from the CRIMEDIM team and collaborating experts, as well as undergraduate and postgraduate students from various programmes. The experts were chosen because of their specialised understanding of the subject matter or their contributions to the development and implementation of the training programme. The experts had a collective expertise profile encompassing disaster medicine, humanitarian health, public health, management of global health education and training programmes, e-learning, and instructional design. All participants recruited, who were students DisasterSISMthe Training Disaster Medicine Trainers (TdmT)the European Master of Science in Disaster Medicine (EMDM)of DisasterSISM, the Training Disaster Medicine Trainers (TdmT), the European Master of Science in Disaster Medicine (EMDM), and Master of Public Health (MPH) were eligible to participate in the study, as they represent the target audience of the SBES. The participants were contacted through emails initially, since it was during the COVID-19 period. Further communication through the action cycles took place either online or face to face based on their availability. Those who consented to participate in the study were recruited. While the formative evaluation involved both experts and students, the summative evaluation involved CRIMEDIM students at different levels.

For quantitative evaluation, we targeted all the students from TdmT, 2022, to represent the undergraduate and all students from EMDM, 2022, to represent postgraduate trainees. Out of the total 57 students enrolled in the 2022 academic year (22 enrolled in TdmT and 35 in EMDM), 35 (61.4%) gave consent to the evaluation feedback and submitted the multiple-choice questions (MCQs) assessment.

### Training development framework

A modified version of the analysis, design, development, implementation and evaluation model, known as rapid prototyping, was used for SBES development.^36 37^ This process involved iterative cycles of formative and summative evaluations.

### Part A: SBES development

#### Analysis phase

The analysis phase involved reviewing literature and training programmes in humanitarian health,^4^ ^38^ alumni surveys^39^ and review of CRIMEDIM training curricula, and stakeholders’ feedback.

#### Design and development phase

Findings of the analysis phase were used to identify and prioritise the learning outcomes, training content and instructional design characteristics.^4 40^ Bloom’s taxonomy was used to guide development of the learning outcomes.^41^ The Sphere handbook and literature review guided scenario script writing.^40^ The adult learning and experiential learning theories^42 43^—including the learning theories of behaviourism, cognitivism, constructivism, connectivism and their derivatives^44^,^47^—guided the design of SBES characteristics and activities. The modules and graphics were developed using Articulate Storyline 360PowerPoint for Microsoft Office 365Articulate Storyline 360, PowerPoint for Microsoft Office 365 andAdobe Illustrator CC Adobe Illustrator CC software, 2019. The assessment tool was developed by partners from the Centre for Research on Healthcare in Disasters at Karolinska Institutet, guided by the intended learning outcomes. The SBES design was tailored based on the stakeholders’ feedback.

#### Implementation phase

Rapid prototyping involved delivering the developed SBES modules to students through learning management systems within relevant existing modules/or residential phases of their training programmes, while experts were provided with the link to articulate review, where they can use and comment directly on the SBES. Participants were invited to complete the self-paced stand-alone SBES and provide feedback.

#### Action

Characteristics of the SBES prototype that received a rating of three or less on a five-point numerical scale were evaluated and modified. All suggestions for incorporation into the training were addressed within the existing resources and incorporated into subsequent module development. In case of disagreement among stakeholders, we prioritised integrating suggestions based on their relevance to the implementation purpose for the specific group (experts or students), their alignment with the purpose of the training, the frequency with which they were reported, and their adherence to documented best practices in e-learning development.

### Part B: SBES evaluation

The formative evaluation aimed to provide continuous feedback during the SBES development process to adjust and improve SBES quality. The summative evaluation phase aimed to validate the effective SBES design elements, which were deemed valuable for learning from the perspective of different stakeholders, and to assess the SBES training effectiveness by measuring the first two levels of the Kirkpatrick model: students’ reactions and students’ learning.^48 49^

#### Data collection methods and tools

##### Qualitative data collection

During the formative evaluation, the principal investigator and the CRIMEDIM team had face to face and online interactions. The students primarily interacted online with occasional face to face meetings. The interaction with CRIMEDIM collaborators and Public Health Masters students was entirely online.

Students provided feedback through post-training anonymised questionnaires, which included closed-ended and open-ended questions. The questionnaire focused on various aspects of SBES, such as structure, content, quizzing, navigation, visual design and interactivity.^50^ Modified versions of the questionnaire were used in different cycles based on emerging findings. Some students also provided feedback via email and WhatsApp.

Experts also provided feedback through an anonymous questionnaire, articulate review (where comments were shared and discussed among reviewers), and informal unstructured interviews guided by the questionnaire themes. Additionally, stakeholders shared their perspective on effective SBES design by highlighting the aspects they found most valuable.

When saturation was achieved, and no new insight emerged, all qualitative data collected during the formative and summative evaluations were analysed and presented.

##### Quantitative data collection

Student reaction was measured using a post-training survey. The survey was developed from the thematic framework that emerged from the formative evaluation phase. The survey measured student reaction towards SBES quality and satisfaction on a 1–7 numerical scale with 18 questions covering four domains: instructional content, graphics and multimedia, design and technology, and overall experience (online supplemental material 1).

A reliability analysis check was carried out on the scale and showed excellent internal consistency (Cronbach’s α=0.968).^51^ A quasi-experiment was conducted to measure the change in the students’ knowledge score post-test compared with pre-test.

### Data analysis

#### Qualitative analysis

The analysis and interpretation of the feedbacks followed qualitative iterative thematic inquiry as recommended by Morgan and Nica.^52^ The initial phase of data analysis started before data collection by drafting a description of the themes, which are commonly used for e-learning evaluation.^50^ Feedback memos, detailing stakeholders' perspectives on existing problems and potential solutions, were assessed to determine their implications, and appropriate corrective measures were implemented. Furthermore, new insights within the collected data emerged during data collection and were used to inform data collection of the next phase of implementation.

Themes that emerged regarding the SBES effective design elements were revised and listed into a thematic framework, and a coding system was developed and applied to the qualitative data. The emerging themes were also used as a framework to develop a questionnaire that measured students’ reactions quantitatively during post-SBES training.

#### Quantitative analysis

The quantitative data were described using percentages for categorical data or mean for continuous data and intervals, including the numerical rating scale.^53^ The 95% CI was reported for continuous data and proportions with binomial distribution. The analysis of each question was based on the total number of respondents per question. To assess students’ learning, a paired t-test was used to assess if the post-test knowledge score has changed significantly compared with the pre-test knowledge score. The tests were performed with IBM SPSS V.29 (IBM Corp.; Armonk, New York, USA). A value of p<0.05 was considered a statistically significant result. An effect size >0.8, which is of high effect, was considered as the desired outcome.^54^

### Reflexivity

Action research requires the researcher to actively participate in achieving the desired outcome of the study. The principal investigator (AB) initiated an action-design project at CRIMEDIM in close collaboration with LR, the scientific coordinator of the center.

The principal investigator acknowledges that her previous professional career as a public health lecturer and practitioner in a humanitarian setting, as well as LR’s current position and his experience as disaster medicine professor with international field experience, may have influenced their research experiences and impacted the design, development and presentation of this study.

To mitigate potential biases associated with action research, various stakeholders were involved throughout different cycles of the study. These stakeholders included the CRIMEDIM team, external experts, and students at different levels within and outside CRIMEDIM. Their involvement aimed to explore and triangulate the results from different perspectives. Additionally, different methods were employed to collect data from various sources and stakeholders using both direct and anonymised forms. Furthermore, an external collaborator developed the MCQs assessment to minimise potential biases associated with the assessment tool development.

### Patient and public involvement

None.

## Results

### Part A: SBES development

#### Analysis phase

Health need assessment, health services delivery and health system emerged as essential topics in humanitarian health. Different levels of training are required. The formative evaluation also revealed that most of the targeted students lack basic knowledge of public health in humanitarian crises. Based on these findings, the developed training was designed to target health professionals who are new to or have limited experience in the humanitarian field. Additionally, conceptual simulation such as SBES was identified as an appropriate instructional strategy to address the learning objectives of the identified topics through e-simulation.

#### Design and development phase

Measurable learning outcomes, based on competencies identified from analysis of the Sphere minimum standard in health, were developed using Bloom's taxonomy (table 1). Four SBES modules were developed to address the needs. The learning outcome and SBES design have been refined and modified based on the stakeholders’ feedback. See box 1 for a detailed description of the developed SBES modules.

**Table 1:** Humanitarian health action scenario-based e-simulation

Domain: Public Health (health system and health services during humanitarian crises)
Aim: To learn about the humanitarian health standard in order to implement appropriate health strategies and interventions during a humanitarian response.

Competency	Topic	Learning Outcomes
To understand the health needs of the affected population and approaches/guidelines to deal with them. To understand the components of a functional health system, including the types of health services. To understand how health system components and services are established and modified. To identify the different technical areas in public health response in complex humanitarian emergencies (CHEs). To develop health interventions and strategies, responsive to the cultural values of the community being served, tailored to time and resources.	Health needs assessment	Identify priorities for health needs assessment. Define important data to collect. Identify sources and methods for data collection. Evaluate strengths and limitations of data within a humanitarian context. Prioritise interventions based on health needs assessment.
	Essential health services	Identify the standards of essential health services. Select appropriate strategies to address the immediate humanitarian needs. Recognise the challenges of providing healthcare during a crisis. Collaborate with different stakeholders.
	Communicable diseases	Identify the minimum health standards in communicable diseases. Prioritise health interventions based on health needs. Perform outbreak initial investigation. Calculate attack rate, case fatality rate.
	Health system	Identify the building blocks of the health system. Recognise the challenges of health systems in humanitarian settings. Identify the different stakeholders of the health systems. Discuss interventions to strengthen the health systems.

Box 1 **Scenario-based e-simulation (SBES) in humanitarian health action**

The SBES for humanitarian health action has four modules targeting health professionals at the entry level of the humanitarian field. All modules are based on a case study of a fictional country suffering from a protracted crisis. Each module has additional scenario(s) to the country’s original scenario to reflect the learning goals of the module. The student plays the role of a health programme manager in a non-governmental organisation and encounters several tasks, where s/he needs to take a decision, solve a problem or explore the situation.

The tasks are usually in a form of multiple-choice questions, drag-and-drop options or calculations followed by feedback that allows for self-evaluation of key objectives. Moreover, the feedback also provides an opportunity for ‘decontextualisation’ in order to construct generalisable knowledge for the humanitarian context. Other tasks include the exploration of concepts, stakeholders, standards, guidelines and resources relevant to the humanitarian context through interaction of different multimedia. Identifying and interacting with health stakeholders to address several issues was central to the learning objectives of all modules.

The modules were delivered in English, and a closed caption feature was added to enable international students to comprehend the audio content.

The first module is entitled ‘Health needs assessment’. It explores priorities and challenges for conducting a health need assessment during a protracted crisis.

The second module, ‘Essential health services’ explores the strategies and challenges for providing essential services for crisis-affected populations.

The third module, ‘Communicable diseases standards’ is related to the second scenario and deals with the standards of communicable diseases services in a humanitarian crisis.

The fourth module, ‘Health system’ introduces trainees to the concept of health systems, their building blocks, and strategies and challenges for health systems strengthening interventions in humanitarian settings.

#### Implementation phase

A total of 51 participants provided feedback during the formative evaluation. The responses evaluated the content and simulation characteristics for effective SBES from the perspectives of students and experts, to improve the existing module and develop the next module. The first implemented version targeted experts with few students, while the subsequent versions targeted both students and experts, with an increasing number of students involved in each implementation. A summary of the implementation phase is provided in online supplemental material 2.

### Part B: SBES evaluation

#### Qualitative results

##### SBES effective design elements

**Figure 1:**
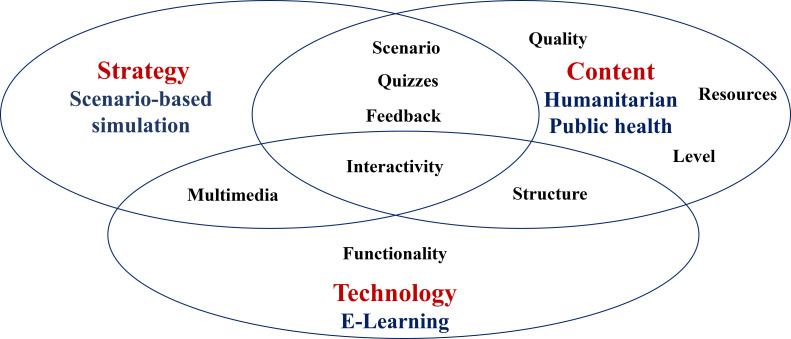
Effective design elements of scenario-based e-simulation (SBES).

Two overarching themes (content and simulated element) and 3 overlapping subthemes, subdivided into 10 overlapping categories for designing effective SBES, emerged from qualitative data analysis (figure 1).

Overall, stakeholders were satisfied with the SBES as a teaching method including the students who were not satisfied with the content, *“I don't feel like I learned a lot through the exercise. I think there is potential in this”—postgraduate student*. Students believed *“it was a very interesting approach to a hard topic, using E-simulation”—postgraduate student,* and which provided them *“with new insight to disaster medicine beyond readings!!”—medical student*. Many students demanded providing more topics within humanitarian health and disaster medicine using e-simulation*: “I would recommend providing more of these types of exercises in preparation for the EMDM residential course”—postgraduate student*. While *s*ome students referred to a whole module (health needs analysis, communicable disease, essential health services or health system) as the most valuable aspect of the SBES, other highlighted specific design element(s) that enhanced their learning experience. The following are the summary of design elements, which stakeholders perceived as the valuable aspect of the SBES:

##### Content quality

Respondents valued the SBES content. Students valued the comprehensiveness and quality of the content, including information presented on different topics of their interest as a student noted that the content was *“oriented towards my possible future professional roles”—medical student*. Experts agreed that the content was relevant, and one expert commented that the content was*“useful for any health professional”—expert*. Almost all the reviewers agreed that the objectives were consistent with the content of the training.

##### Content level

They also noted that the content is dependent on the trainee’s background, and it is suitable as an introductory training, for postgraduate health professionals and senior undergraduate medical students, especially those who have previous knowledge on public health. The level of content was an area of disagreement between different respondents, particularly postgraduate trainees. Some postgraduate students were satisfied with the level of the content, others demanded more advanced content, while others needed more explanation of the theoretical basics. Experts agreed that this training serves as a good introduction to humanitrian health field for postgraduate health professionals or senior undergraduate medical students. However, experienced humanitarian workers will need additional advanced training. A student commented *“Well, it would be a little naive to think after completing the assessment tool to be able to perform a field assessment yourself. This can be process with multiple challenges, but it is a nice start to get a general idea about it”—postgraduate student*.

##### Resources

The students also appreciated the list of references and further training opportunities they were linked to. Like the content level, postgraduate students have different opinions regarding the amount of the references, and while one demanded *“more literature”—postgraduate student*, another commented *“too much theoretical reference material. Could help the student with more focused references?”—postgraduate student*.

##### Simulation structure

All reviewers agreed that the content was clear, understandable, logically arranged and well structured and *“mixing theoretical concepts to the example/exercise”*—*expert*, and *“provide[ed] organised way for practice the content”—postgraduate student*. While an expert suggested a more linear structure, another expert suggested a more branching scenario. Revisiting of concept form different angles through different modules was considered valuable by an expert.

##### Simulation scenario

One of the most valued features of SBES design was the practicality and reality of the scenario *“The reality of the exercise content simulates a large part of the health reality required to confront crises and disasters”—postgraduate student*. Experts believe that SBES will allow students to learn *“by doing”—expert,* while SBES enables the student to recognise how all the *“humanitarian responses are arranged within a big frame”—medical students*.

Students also valued the *“full immersion in the process”—medical students*, including the *“the interpretation of a role as it is in real-life when being a humanitarian aid worker, for example, the discussions with the health cluster members to take decisions on what to do”—postgraduate student*. Another student responded to the most valuable aspect, *“For me, it was the role, I think that we feel the responsibility, and that makes us engage more with the results”—medical students*.

##### Quiz and feedback

The most valuable features of the SBES were the quiz and the immediate constructive feedback, which has been mentioned frequently by all reviewers. Besides increasing interactivity, this strategy allowed the students to test, evaluate and reflect on their own knowledge, misconception and common malpractice. An expert commented *“I think the quizzes are a good way to make people think practically and learn from their response and later feedback”—expert*. Similarly, a student also commented *“It really helped to focus on the mistakes, and prevent them from happening”—postgraduate student*.

##### Multimedia

They also valued simplicity, reader-friendliness, clarity, and level of realistic details within the multimedia presented, which also reflect the content including the images that *“gave a picture of the content without reference to speech and writing, very nice”—postgraduate student*, while another student commented *“I was fascinated by the amount of details (click the message, see the report, the maps, etc)”—medical students*.

##### Interactivity

Students valued the interactivity of SBES, and one commented *“It is interesting to be active to react”—postgraduate student,* another student *responded, “Loved the course and its interactivity ” —medical students*.

##### Functionality

Experts and students valued SBES’s simplicity and ease of access and use. Students also appreciated the autonomy/individuality associated with SBES, being *“able to proceed at your own pace and repeat whenever necessary.”—medical students*.

### Quantitative results of SBES effectiveness

#### Reaction

The majority of the participants did not have any previous training or experience in public health or humanitarian health (68.6%, 95% CI 52.2% to 82%; n=24/35), while 25.7% (95% CI 13.6% to 41.7%;n=9/35) of the participants (mainly undergraduate students (n=7)) had both public health and humanitarian health training.

On a 1–7 numerical rating scale, the mean rating of respondents for the SBES design element was 5.95 (95% CI 5.62 to 6.25), and the mean rating for overall satisfaction with SBES’s learning experience was 6.03 (95% CI 5.95 to 6.47). See the figure in online supplemental material 2 for further details. Undergraduate students have higher mean satisfaction with the SBES design (mean=6.12, 95% CI 5.77 to 6.47) and SBES learning experience (mean=6.28, 95% CI 5.83 to 6.72) compared with postgraduate students (mean=5.76, 95% CI 4.96 to 6.57 and mean=5.76, 95% CI 5.19 to 6.30, respectively). No statistically significant difference between both groups was found (p=0.238 and p=0.244).

#### Learning

Overall, the mean of the post-test was 7.71, which was significantly higher than the mean of the pretest, which was 5.88 (p<0.001). The large effect size of 1.179 indicates the effectiveness of SBES training.

There was no statistically significant difference (*p*=0.842) in the score of the pretest between undergraduates (n=17, mean=4.94, SD=2.35) and postgraduates (n=18, mean=4.82, SD=1.18). However, the scores of the post-test undergraduates (n=17, mean=8.63, SD=2.02) and the EMDM (n=18, mean=6.85, SD=1.37) are significantly different between both groups (*p*=0.005). This result indicates that the highest change in the scores achieved after the intervention was among undergraduate students. For more details, see the figures in online supplemental material 2.

## Discussion

Despite the upward trend in humanitarian crises,^55^ humanitarian health education and training programmes are still limited, unequally distributed and dominated by theoretical teaching methods.^4^ To fill this training gap and align it with global demands, we employed mixed-methods action research and an iterative process involving diverse stakeholders for SBES development and evaluation. Four modules targeted at entry-level health professionals were developed: health needs assessment; essential health services; communicable diseases; and health system.

The SBES evaluation showed a high satisfaction rate and significant improvement in knowledge score. These findings suggest that SBES addresses the academic centre training gaps and has practical implications for enhancing accessibility and teaching methods, particularly in resource-limited settings. In addition, the study identified the design elements of SBES, which contribute to the theoretical understanding of SBES^28^ and hold implications for future SBES design and evaluation. The findings also suggest that the iterative design process incorporating perspectives from multiple stakeholders and informing SBES design by learning theories enhances learning satisfaction and outcomes among students.

The elements of SBES design, identified through evaluation, were aligned with simulation-related learning theories and the simulation concept analysis proposed by Bland *et al*.^56^ For instance, SBES activities were outcome-based, akin to behaviourism, and highly valued by both students and experts. Students also appreciated the ability to revisit the SBES practice as needed.^44^,^4656 57^ In line with connectivism, students expressed satisfaction when they were connected with additional learning resources through the SBES.^44 58^ Authentic reality recreation through realism, visuals and real-life tasks is aligned with cognitivism.^44^,^46^ The spiral content design allowed students to build on their existing knowledge.^59^ Students also frequently mentioned problem-solving tasks and immediate feedback as the most useful design elements,^29 58 60^ allowing them to reflect, construct new knowledge^44^,^4659^ and evaluate the pre-existing one.^44^

The role of technology was pivotal in ensuring SBES’s fidelity and interactivity;^61 62^ SBES’s proper design and functions are crucial for a positive learning experience. However, technology can be a barrier to learning. For example, poor internet connectivity posed challenges for Yemeni students in accessing and completing the training. To mitigate this issue, an offline version of the SBES should be developed. Furthermore, broader barriers, such as technological readiness, necessitate attention, particularly in humanitarian and global health contexts. Hence, a comprehensive evaluation framework for e-learning has emerged as imperative, extending beyond the tool itself.^63^

Interestingly, undergraduates had higher response rates and exhibited significant post-test score improvements compared with postgraduates, although both groups had similar pretest scores. These distinctions were further evident in the formative evaluation, particularly regarding the level of training content, in which postgraduates exhibited more varied responses. This result could be due to the diverse educational backgrounds and experience levels. Moreover, postgraduates may lack motivation due to their established career paths that may not align with the humanitarian response,^64^ or they might face time constraints due to work and personal commitments. Investigating the underlying reasons for these differences would facilitate the adaptation of SBES to accommodate the diverse needs of students. Nevertheless, the evaluation suggested potential benefits for both undergraduate and postgraduate students.

Although the study showed satisfactory results, it had limitations. The action research design limits generalisation of findings beyond the specific academic context, particularly for SBES effectiveness, which evaluated satisfaction and knowledge only. Since the SBES, including its content, was developed during the study period, no other training modality was available for comparison. Hence, the evaluation was limited to a single-group quasi-experimental design, which has low internal validity but indicates real-world behaviour. Nevertheless, the study primarily aimed to meet the training needs of an international academic centre, thus holding practical contextual relevance. Future research should explore the transferability of the SBES approach to diverse educational contexts and evalautes its outcome at different levels to determine its broader effectiveness and applicability.

Additionally, addressing the diverse needs of different stakeholders within the constraints of time and resources is challenging. Furthermore, engaging external collaborators proved difficult, primarily because of the COVID-19 pandemic, resulting in a limited number of external stakeholders participating in the study. Despite this, training with external MPH students from Yemen yielded consistent findings on the SBES design. It is also important to note that the design of SBES, driven by the academic centre requirement for a stand-alone tool, limited the scope of human social interaction, which could have potentially enhanced the simulation learning experience.

## Conclusion

Through the successful design and evaluation of SBES, this study effectively addressed gaps in accessibility and teaching methods in humanitarian health education by aligning identified needs of the training center with global demand. The developed SBES demonstrated effectiveness, as evidenced by students’ positive reactions and significant improvement of post-test knowledge score. Therefore, it meets the essential needs of humanitarian health professionals. The identified design elements can inform the development and evaluation of advanced training, and other related topics in the field of global health. The study also sheds light on the need for further participatory research and targeted actions to address emerging needs and develop tailored e-learning. Specifically, it highlights the importance of comprehensive evaluation of e-learning methods and their implementation in resource-limited settings, including areas with limited internet access.

## supplementary material

10.1136/bmjopen-2023-079681 online supplemental file 1

10.1136/bmjopen-2023-079681 online supplemental file 2

## Data Availability

Data are available upon reasonable request.

## References

[1] United nations office for the coordination of humanitarian affairs (2018). World humanitarian data and trends 2018.

[2] Jawad M, Hone T, Vamos EP (2020). Estimating indirect mortality impacts of armed conflict in civilian populations: panel regression analyses of 193 countries, 1990-2017. BMC Med.

[3] Niescierenko M, Fischer HT, Prager G (2019). Strengthening global capacity for emergency health action. https://healthcluster.who.int/publications/m/item/strengthening-global-capacity-for-emergency-health-action.

[4] Bahattab AAS, Linty M, Trentin M (2022). Availability and characteristics of humanitarian health education and training programs: a web-based review. Prehosp Disaster Med.

[5] Kalbarczyk A, Harrison M, Sanguineti MCD (2020). Practical and ethical solutions for remote applied learning experiences in global health. Ann Glob Health.

[6] Manning ML, Jack D, Wheeler L (2022). Effect of a virtual simulated participant experience on antibiotic stewardship knowledge among pre-licensure baccalaureate nursing students: a pilot study. Nurse Educ Today.

[7] Kivlehan SM, Tenney K, Plasmati S (2022). Humanitarian training with virtual simulation during a pandemic. Disaster Med Public Health Prep.

[8] Kalbarczyk A, Nagourney E, Martin NA (2019). Are you ready? A systematic review of pre-departure resources for global health electives. BMC Med Educ.

[9] Nadeau C, Snowden K, Gattamorta KA (2020). Use of simulation for global health pre-departure training. Nurse Educ Today.

[10] Bertelsen NS, DallaPiazza M, Hopkins MA (2015). Teaching global health with simulations and case discussions in a medical student selective. Glob Health.

[11] Mohamed-Ahmed R, Daniels A, Goodall J (2016). ‘Disaster day’: global health simulation teaching. Clin Teach.

[12] Tartari E, Fankhauser C, Peters A (2019). Scenario-based simulation training for the WHO hand hygiene self-assessment framework. Antimicrob Resist Infect Control.

[13] Reina Ortiz M, Sharma V, Casanova J (2021). Developing global health diplomacy-related skills using a COVID-19-like epidemic simulation as a learning strategy. Am J Trop Med Hyg.

[14] Wong BLH, Khurana MP, Acharya N (2020). World health organization simulations: an increasingly popular learning tool for the development of future global health practitioners. J Glob Health.

[15] French AJ, Masys AJ, Izurieta R, Ortiz MR (2020). Global health security: recognizing vulnerabilities, creating opportunities, aadvanced sciences and technologies for security applications.

[16] Kawosa B, Miller ET (2019). Comparative cost of virtual reality training and live exercises for training hospital workers for evacuation. Comput Inf Nurs.

[17] Bustamante ND, Rouhani SA, Kivlehan S (2020). The Haiti humanitarian response course: a novel approach to local responder training in international humanitarian response. Prehosp Disaster Med.

[18] Williams H, Downes E (2017). Development of a course on complex humanitarian emergencies: preparation for the impact of climate change. J Nurs Scholarsh.

[19] Dickey C, Holzman E, Bedford J (2021). Behavioral communication strategies for global epidemics: an innovative model for public health education and humanitarian response. Health Promot Pract.

[20] Ripoll-Gallardo A, Ragazzoni L, Mazzanti E (2020). Residents working with Médecins sans frontières: training and pilot evaluation. Scand J Trauma Resusc Emerg Med.

[21] Bajow NA, Alawad YI, Aloraifi SM (2019). A basic course in humanitarian health emergency and relief: a pilot study from Saudi Arabia. Prehosp Disaster Med.

[22] Nouvet É (2016). Beyond doing good: an interview with Dr. Kirsten Johnson on the Canadian disaster andhumanitarian response training program. BioethOnline.

[23] Varpio L, Bader Larsen K, Hamwey M (2021). Interprofessional education in the U.S. military: harnessing simulation for team readiness. J Interprof Care.

[24] Evans AB, Hulme JM, Nugus P (2017). An electronic competency-based evaluation tool for assessing humanitarian competencies in a simulated exercise. Prehosp Disaster Med.

[25] Cranmer H, Chan JL, Kayden S (2014). Development of an evaluation framework suitable for assessing humanitarian workforce competencies during crisis simulation exercises. Prehosp Disaster Med.

[26] Bodas M, Peleg K, Adini B (2022). Training package for emergency medical TEAMS deployed to disaster stricken areas: has ‘TEAMS’ achieved its goals?. Disaster med public health prep.

[27] Istrate O, Kestens A (2015). The 11th international scientifc conference elearning and software education.

[28] Bajpai S, Semwal M, Bajpai R (2019). Health professions’ digital education: review of learning theories in randomized controlled trials by the digital health education collaboration. J Med Internet Res.

[29] Clark RC, Mayer RE (2011). E-Learning and the science of instruction: proven guidelines for consumers and designers of multimedia learning.

[30] Battista A (2017). An activity theory perspective of how scenario-based simulations support learning: a descriptive analysis. Adv Simul (Lond).

[31] Kageyama Y, Zamudio SZ, Barton M (2022). Incorporation of simulation features to improve higher order thinking skills. Int J Manag Educ.

[32] Van Hecke A, Duprez V, Pype P (2020). Criteria for describing and evaluating training interventions in healthcare professions – CRe-DEPTH. Nurse Educ Today.

[33] McKenney S, Reeves TC (2021). Educational design research: portraying, conducting, and enhancing productive scholarship. Med Educ.

[34] Oberschmidt K, Grünloh C, Nijboer F (2022). Best practices and lessons learned for action research in eHealth design and implementation: literature review. J Med Internet Res.

[35] Ivankova N, Wingo N (2018). Applying mixed methods in action research: methodological potentials and advantages. Am Behav Sci.

[36] Daugherty J, ting TY, Cornachione E (2007). Rapid prototyping instructional design: revisiting the ISD model. Pap Present Int Res Conf Am Acad Hum Resour Dev.

[37] Piskurich GM (2015). Rapid instructional design: learning ID fast and right.

[38] Bahattab A, Trentin M, Hubloue I (2024). Humanitarian health education and training state-of-the-art: A scoping review. Front Public Health.

[39] Bahattab AAS, Linty M, Hubloue I (2022). The involvement of the European Master in Disaster Medicine (EMDM) alumni in the COVID-19 pandemic response: an example of the perceived relevance of disaster medicine education during disasters. Prehosp Disaster Med.

[40] Sphere Association (2018). The sphere handbook: humanitarian charter and minimum standards in humanitarian response.

[41] Adams NE (2015). Bloom’s taxonomy of cognitive learning objectives. J Med Libr Assoc.

[42] Knowles MS, Holton III EF, Swanson RA (2005). The adult learner: the definitive classic in adult education and human resource development.

[43] Kolb DA, Fry R, Cooper IC (1975). Towards an applied theory of experiential learning.

[44] Babin MJ, Rivière CG, Katsaropoulos C (2019). Theory for practice: learning theories for simulation.

[45] D. Erlam G, Smythe L, Wright-St Clair V (2017). Simulation is not a pedagogy. OJN.

[46] Taylor L (2004). Educational theories and instructional design models: their place in simulation. Educ Theor Instr Des Model Their Place Simul.

[47] Bahattab A, Caviglia M, Martini D (2023). Scenario-based e-simulation design for global health education: theoretical foundation and practical recommendations. J Med Internet Res.

[48] Kirkpatrick DL, Craig RL, Bittel LR (1970). Evaluation of traning.

[49] Johnston S, Coyer FM, Nash R (2018). Kirkpatrick’s evaluation of simulation and debriefing in health care education: a systematic review. J Nurs Educ.

[50] Wood SJ, Rogers MH, Frost MC (2019). Enhancing access to quality online training to strengthen public health preparedness and response. J Public Health Manag Pract.

[51] Taber KS (2018). The use of cronbach’s alpha when developing and reporting research instruments in science education. Res Sci Educ.

[52] Morgan DL, Nica A (2020). Iterative thematic inquiry: a new method for analyzing qualitative data. Int J Qual Methods.

[53] Treiblmaier H, Filzmoser P (2011). Benefits from using continuous rating scales in online survey research. Int Conf Inf Syst.

[54] Sullivan GM, Feinn R (2012). Using effect size-or why the P value is not enough. J Grad Med Educ.

[55] United Nations Office for the Coordination of Humanitarian Affairs (2022). Global humanitarian overview 2023.

[56] Bland AJ, Topping A, Wood B (2011). A concept analysis of simulation as a learning strategy in the education of undergraduate nursing students. Nurse Educ Today.

[57] Nestel D, Bearman M (2015). Theory and simulation-based education: definitions, worldviews and applications. Clin Simul Nurs.

[58] Cook DA, Hatala R, Brydges R (2011). Technology-enhanced simulation for health professions education: a systematic review and meta-analysis. JAMA.

[59] Bensadon BA, Deering S, Auguste TC, Goffman D (2019). Comprehensive healthcare simulation: obstetrics and gynecology.

[60] Issenberg SB, McGaghie WC, Petrusa ER (2005). Features and uses of high-fidelity medical simulations that lead to effective learning: a BEME systematic review. Med Teach.

[61] Sandars J, Patel RS, Goh PS (2015). The importance of educational theories for facilitating learning when using technology in medical education. Med Teach.

[62] Mayer RE, Mayer RE (2005). Cognitive theory of multimedia learning.

[63] Bahattab A, Hanna M, Teo Voicescu G (2023). E-learning evaluation framework and tools for global health and public health education: protocol for a scoping review. JMIR Res Protoc.

[64] Da Silva GMC, Borges AR, Da Silva Ezequiel S (2018). Comparison of students’ motivation at different phases of medical school. Rev Assoc Med Bras.

