# Supplementary material for: Development and evaluation of scenario-based e-simulation for humanitarian health training: a mixed-methods action research study
Source: BMJ Open. 2024 Aug 5;14(8):e079681. doi: 10.1136/bmjopen-2023-079681 (PMC11308908; doi:10.1136/bmjopen-2023-079681)
Supplement: online supplemental file 1 [file bmjopen-14-8-s001.pdf]

Supplementary document (II) Summative Evaluation

Humanitarian Health Action exercises feedback survey

Kindly take a few minutes to complete this anonymous questionnaire.
Please note that this questionnaire is not a student evaluation but an important part of the exercise delivery academic review. We take your anonymous comments very seriously, which will significantly influence the way that we improve the modules and programs over time. Thank you for your time and cooperation.

Consent

If you consent, the data you provided may be used anonymously for research purposes.

Do you consent that the data you provided can be used for research purposes?
- Yes
- No

A. Instructional content

Please rate how much do you agree with the following statements, which describe the humanitarian health exercises feature: (Seven (7) means you strongly agree that the exercises have that feature, and a one (1) means you strongly disagree)

1. The exercises provide learning content that I need

Strongly disagree 1 2 3 4 5 6 7 ***Strongly agree***

2. The exercises provide me with sufficient learning content

Strongly disagree 1 2 3 4 5 6 7 ***Strongly agree***

3. The contents of the exercises are useful to my current/expected professional role

Strongly disagree 1 2 3 4 5 6 7 ***Strongly agree***

4. The contents of the exercises are easy to understand

Strongly disagree 1 2 3 4 5 6 7 ***Strongly agree***

5. The contents of the exercises are logically organized

Strongly disagree 1 2 3 4 5 6 7 ***Strongly agree***

6. The content of the exercises are clearly related and applicable to real-world situations

Strongly disagree 1 2 3 4 5 6 7 ***Strongly agree***

7. The virtual scenario (e.g. Maurania) helped me to gain a clearer understanding of the content

Strongly disagree 1 2 3 4 5 6 7 ***Strongly agree***

8. The quiz feedback process helps to gain new knowledge

Strongly disagree	1	2	3	4	5	6	7	***Strongly agree***

9. The exercises have good interactivity

Strongly disagree	1	2	3	4	5	6	7	***Strongly agree***

10. The exercises provide relevant additional resources and learning material

Strongly disagree	1	2	3	4	5	6	7	***Strongly agree***

B. Graphic and multimedia

Please rate how much do you agree with the following statements, which describe the humanitarian health exercises feature: (Seven (7) means you strongly agree that the exercises have that feature, and a one (1) means you strongly disagree)

1. The exercises use audio elements properly

Strongly disagree	1	2	3	4	5	6	7	***Strongly agree***

2. The exercises use animations/graphics features properly

Strongly disagree	1	2	3	4	5	6	7	***Strongly agree***

3. The exercises use multimedia features properly

Strongly disagree	1	2	3	4	5	6	7	***Strongly agree***

C. Design and technology

Please rate how much do you agree with the following statements, which describe the humanitarian health exercises feature: (Seven (7) means you strongly agree that the exercises have that feature, and a one (1) means you strongly disagree)

1. The exercises are well structured

Strongly disagree	1	2	3	4	5	6	7	***Strongly agree***

2. The exercises navigation is easy to use

Strongly disagree	1	2	3	4	5	6	7	***Strongly agree***

D. Satisfaction and overall experience

Please rate how much do you agree with the following statements, which describe the humanitarian health exercises feature: (Seven (7) means you strongly agree that the exercises have that feature, and a one (1) means you strongly disagree)

1. Overall, my experience with the exercises has been enjoyable

Strongly disagree	1	2	3	4	5	6	7	***Strongly agree***

2. Overall, the quality of the exercises is excellent

Strongly disagree	1	2	3	4	5	6	7	***Strongly agree***

3. Overall, I am satisfied with the exercises

Strongly disagree	1	2	3	4	5	6	7	***Strongly agree***

- What aspects of this exercise were most useful or valuable?

Strongly disagree	1	2	3	4	5	6	7	***Strongly agree***

- How would you improve this exercise?

Strongly disagree	1	2	3	4	5	6	7	***Strongly agree***

Background information

1. Which of the following better describe your professional background:
   - Physician
   - Nurse/allied health professionals
   - Other
2. Do you have previous training and/ or experience in public health?
   - Yes
   - No
3. Do you have previous training and/ or experience in humanitarian health?
4. Yes
5. No

Final comment

Are there any final comments? Please feel free to share them below
