# Supplementary material for: Development and evaluation of scenario-based e-simulation for humanitarian health training: a mixed-methods action research study
Source: BMJ Open. 2024 Aug 5;14(8):e079681. doi: 10.1136/bmjopen-2023-079681 (PMC11308908; doi:10.1136/bmjopen-2023-079681)
Supplement: online supplemental file 2 [file bmjopen-14-8-s002.pdf]

Supplementary Tables:

Summary of the implementation cycles

<table>
<tr><th>Implementation</th><th>Participants</th><th>Duration</th><th>Methods (no. of participant)</th><th>Feedback and suggestions</th><th>Actions</th></tr>
<tr><td colspan="6">First Cycle: HHA Module (HNA + CD) version
Aim: to validate content needs, level, instructional design effective design elements functionality and overall experience.</td></tr>
<tr><td>1</td><td>Experts
(CRIMEDIM collaborators) /CRIMEDIM Team</td><td>2 Sep 2020 – 1 October</td><td>Feedback form (n=9)
Articulate review (n=11)
Informal communication (n=4)</td><td rowspan="4">Expert:
• Define the terminologies and abbreviations.
• Add references and additional resources,
• Revise the title and the objectives to match the content.
• Increasing the level of interactivity (add more tasks with feedback, create branching scenarios, and topics, and use interactive multimedia, e.g. different characters with voice-overs).
• Improvement navigation and functionality.
• Cover other topics of health services.
• Typos correction.
• Revise and improve the technical quality of the content material.
• Content difficult without previous reading and acronym explanation compared to senior students.
• Adding a take-home message at the end of the module.
Both experts and students
• Adding introductory information.
• Improve the visual design by adding images to represent the setting.</td><td rowspan="4">• Introductions, take-home message and on-demand glossary, which explains terminologies and abbreviations were added.
• Objectives and content were revised and linked to references and other training opportunities.
• The structure of the module was simplified (by separating different topics within the SBES contents into two separate modules (Health Needs Assessment; Communicable Disease Standards).
• Navigation has been improved by eliminating non-functional and unnecessary clicks.
• SBES interactivity and visual design improved by adding characters with voice-over and animated images that represent the location of the scenario and maintaining colours consistency along each module.
• The Essential Health Service module was developed based on the analysis phase results, as well as the feedback provided during the first cycle.</td></tr>
<tr><td>2</td><td>Undergraduate students (DisasterSISM)</td><td>2 Sep 2020</td><td>Feedback form (n=5)
Informal communication (n=5)</td></tr>
<tr><td colspan="4">First cycle: Updated first Version: HNA, CD</td></tr>
<tr><td>3</td><td>Experts: (CRIMEDIM collaborators, public health experts, Humanitarian, and field practitioners)</td><td>2 Nov 2020 – 30 May 2021</td><td>Feedback form (n=2)
Informal communication (n=5)</td></tr>
</table>

**Second cycle: Three module (HNA, EHS and CDS*) Implementation aim: to validate content needs, level, instructional design effective design elements functionality, implementation feasibility and overall experience for different students' groups.**					
4	CRIMEDIM Team	20 Apr – 1 May 2021	Articulate review (n=5)<br>Informal communication (n=4)	**Students**<br>Provide SBES with multiple languages.<br>Fix technical issues with navigation and audio<br>***CRIMEDIM Undergraduate:*** Add theoretical information before quizzes<br>***CEIMEDIM Postgraduate:*** Variable responses about the content level (Some were satisfied with the level of the content, others demanded more advanced content, while a third group needed more explanation of the theoretical basics).<br>) Adding group tasks and group discussion to increase interactivity, but some,<br>**Postgraduate** (Public health Master- Yemen) reported poor internet connectivity.	• Content was simplified by adding more basic information and expanding the glossary.<br>• Readings and learning resources for both basic and advanced learners were added.<br>• The navigation and functionality issue has been fixed.<br>• More tasks were added to increase the interactivity.<br>• Closed captions were added to help non-English speakers to better follow the voice-over.<br>• The Health System module was developed based on the analysis phase results as well as the feedback provided during the first and second cycles.<br>• Due limitation of resources during the study period, it was not feasible to create additional advanced SBES, provide training in other languages, or facilitate group activities.
5	Postgraduate student disaster medicine (EMDM-CRIMEDIM)	17 May – 30 June	Feedback form (n=15)<br>Informal communication (n=1)		
6	Undergraduate students (DisasterSISM/TdmT -CRIMEDIM)	20 Jul – 15 Jan 2021	Feedback form (n=6)		
7	Postgraduate (Public health Master-Yemen)	1 Dec – 15 Jan 2022	Feedback form (n=5)<br>Informal communication (n=4)		

**Third version: HS module Implementation**<br>**Aim:** To Revise HS module and final check for all module (HNA, EHS, CD, HS)					
8	CRIMEDM team	2 Feb – 1 April 2022	Articulate review (n=4)	Final check for functionality of the Health System module and updated versions of the other modules has been conducted before the training was available to students.	Technical issues of navigation and functionality fixed.
**Final version Implementation: summative evaluation (HNA, EHS, CD*, HS**)**<br>**Aim:** To evaluate SBES effectiveness (student reactions, learning and overall experience)					
9	CRIMEDIM postgraduate students	11 Apr – 15 May 2022	Feedback form (n=17)<br>MCQs test (n=18)	See the results of summative evaluation	
10	CRIMEDIM medical students	15 – 31 Jul 2022	Feedback form (n=18)<br>MCQs test (n=17)		

**HNA: Health needs assessment; EHS: Essential health services, CD: Communicable diseases, **HS: Health system*

Figure S.a.: Summative Evalaution:
Trainees' Reaction to SBES (n=35)

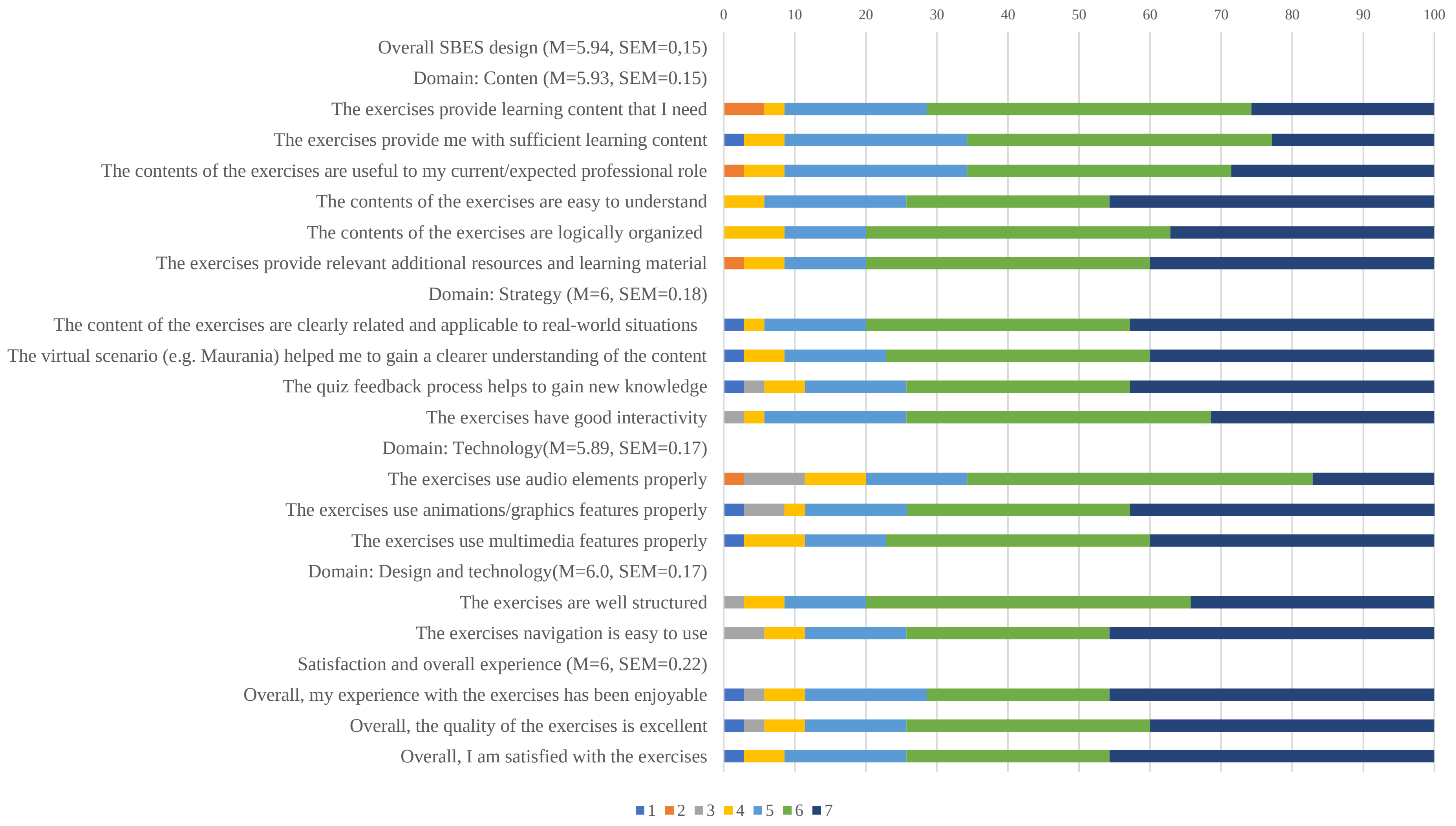

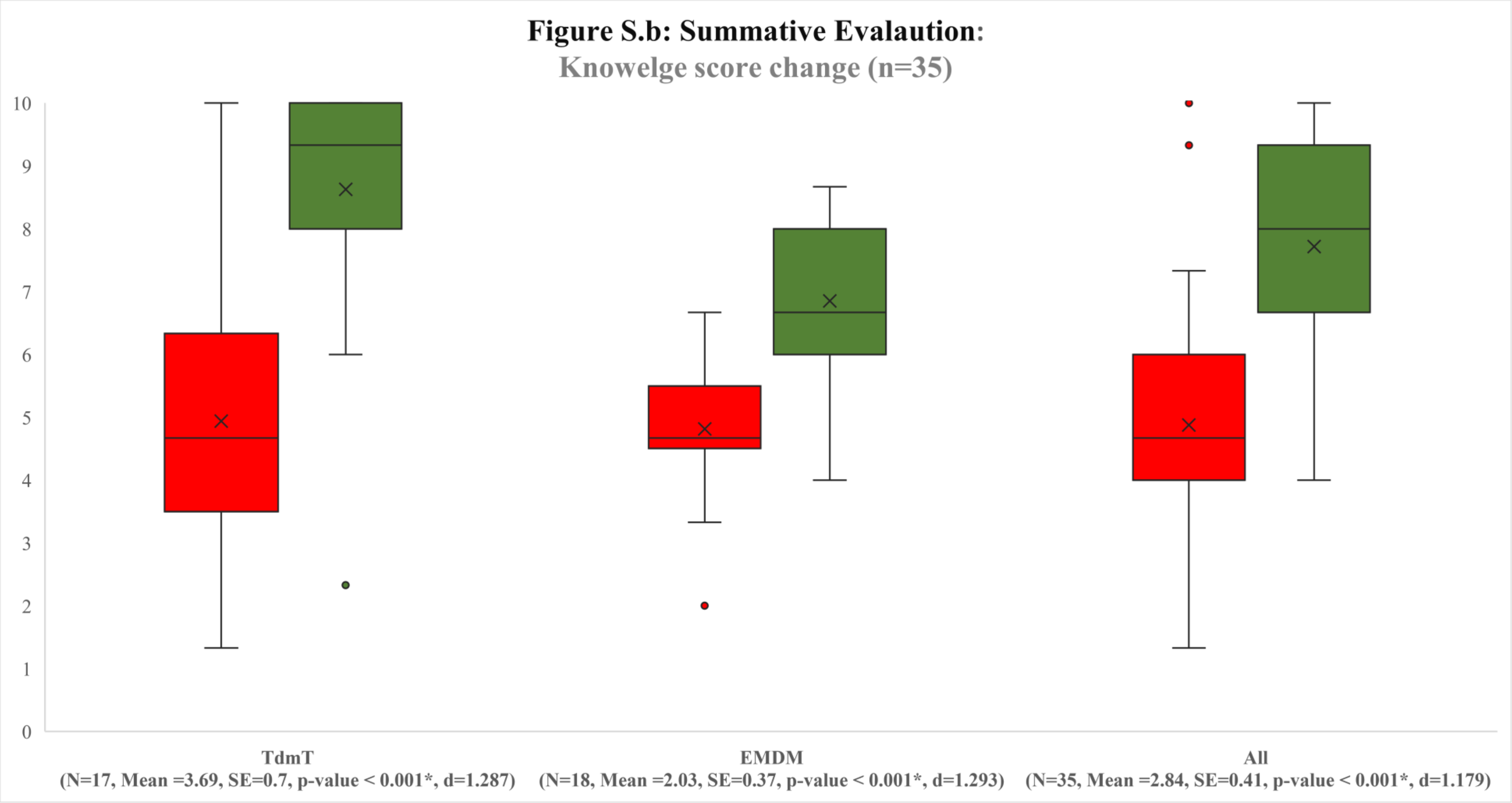
Figure S.b: Summative Evalaution:
Knowelge score change (n=35)
10
9
8
7
6
5
4
3
2
1
0
TdmT
(N=17, Mean =3.69, SE=0.7, p-value < 0.001*, d=1.287)
EMDM
(N=18, Mean =2.03, SE=0.37, p-value < 0.001*, d=1.293)
All
(N=35, Mean =2.84, SE=0.41, p-value < 0.001*, d=1.179)
