# Supplementary material for: Development and evaluation of scenario-based e-simulation for humanitarian health training: a mixed-methods action research study
Source: BMJ Open. 2024 Aug 5;14(8):e079681. doi: 10.1136/bmjopen-2023-079681 (PMC11308908; doi:10.1136/bmjopen-2023-079681)
Supplement: Supplementary file 3 [file bmjopen-2023-079681.reviewer_comments.pdf]

PEER REVIEW HISTORY

BMJ Open publishes all reviews undertaken for accepted manuscripts. Reviewers are asked to complete a checklist review form (**http://bmjopen.bmj.com/site/about/resources/checklist.pdf**) and are provided with free text boxes to elaborate on their assessment. These free text comments are reproduced below.

ARTICLE DETAILS

**TITLE (PROVISIONAL)**	Development and evaluation of scenario-based e-simulation for humanitarian health training: a mixed-methods action research study
**AUTHORS**	Bahattab, Awsan; Zain, Omar; Linty, Monica; Amat Camacho, Nieves; Von Schreeb, Johan; Hubloue, Ives; Della Corte, Francesco; Ragazzoni, Luca

VERSION 1 – REVIEW

**REVIEWER**	Pallavicini, Paolo<br>Vita-Salute San Raffaele University, School of Medicine
**REVIEW RETURNED**	22-Jan-2024

**GENERAL COMMENTS**	Title: Title is interesting, design of the study is not clear enough<br>Abstract:<br>1. Objectives: accessible → accessibility<br>2. You can summarize the objectives in customization and effectiveness<br>Design:<br>Institution-based is too vague. Please summarize at your best the research<br>design.<br>Setting:<br>As per journal guidelines: Generalise; don't use the name of a specific centre<br>but give geographical location if important.<br>Participants:<br>Ok<br>Interventions:<br>There are no interventions made in this study, you already explained that you<br>want to develop and evaluate SBES, no need to add the modules. This part<br>can be omitted.<br>Main outcome measures:<br>Ok<br>Conclusion:<br>Ok<br>FULL PAPER<br>Background:<br>1. "These include … development opportunities" too long, hard to go<br>through.

	2. The introduction is long, 5 paragraph. Make it shorter and more focused on why SBES is important in the humanitarian education setting. The paragraph about the aim is well written.<br>3. Remove CRIMEDIM long title and put it in the setting.<br>Methods:<br>1. I have to admit, I never heard of this research design, but I have analysed the reference 32 and 33, I believe that there are not enough<br>information to conclude that the method used is a modified version,<br>known as rapid prototyping. Rapid prototyping is described different<br>from ADDIE.<br>2. “The study design consist of … strand”, this sentence is vague. A study<br>design must be clear and easy to be repeated.<br>3. “Participants were purposively sampled”.<br>a. Please describe how were they sampled.<br>b. Which were the inclusion criteria?<br>c. How did you reach them?<br>d. Which was the response rate?<br>4. No need to put the footer with the website of the various software<br>used.<br>5. “When saturation was achieved”, how do you define saturation?<br>6. Which software was used for the statistical analysis?<br>7. SBES Development chapter is structured in a way that follow the ADDIE<br>scheme, why the last point is Action and not Evaluation?<br>8. Questionnaire should have been listed as supplementary material.<br>9. You should have put here the (CRe-DEPTH) Checklist.<br>Results:<br>1. Reference should not be present here as you should be just writing the<br>results of your study.<br>2. “However, the formative evaluation ... humanitarian crises” this seems<br>to me more like a discussion point, here you should be listing the results<br>of the formative evaluation.<br>3. SBES effective design validation: this part should be listed as method,<br>together with figure 1<br>4. From “Figure 1: SBES effective design” to “functionality” is a list of<br>comments from the study participants. Feedback and satisfaction rate<br>should be based on appropriate CRF and surveys.<br>5. “Many students valued the interactivity of SBES”, show me how and<br>where, I need to see this in the results.<br>6. SBES Effectiveness: how you recruited the students should be in the<br>methodology,<br>7. “fifty-seven”: In general, words should be used for numbers from zero

	through nine, and numerals should be used from 10 onwards.<br>Discussion:<br>1. “The SBES evaluation showed high satisfaction rate”, how did you<br>measure it?<br>2. You talk about limitations, but you didn’t mention them in the chapter<br>“Strengths and limitations of this study”<br>3. “Hence, the evaluation was limited to a single-group<br>quasi experimental design, which has low internal validity but indicates real world behaviour.” I believe this sentence summarize the reason why this<br>research paper should be a research letter.<br>Conclusion:<br>1. How can you say that you reached a successful design?<br>2. How can you say that this is a bridge, have you explored other interventions?<br>Conclusions should only report facts presented in the results section or<br>discussed in the discussion section.<br>References:<br>1. References Apex should be at the end of a sentence and not in the<br>middle of it.<br>2. I can’t access to the reference number 1, it says “Page not found”,<br>please report access date.<br>3. Reference number 3, please report access date.<br>4. Check reference number 33<br>5. Remove link to reference number 51

**REVIEWER**	Souza, Raissa Silva<br>Universidade Federal de São João del-Rei
**REVIEW RETURNED**	24-Apr-2024

**GENERAL COMMENTS**	Article brings up a topic relevant to continuous education in the healthcare field. The text thoroughly presents the stages of study development, along with results and discussion that shed light on the addressed issue.

VERSION 1 – AUTHOR RESPONSE

Reviewer comments:
Dear Awsan,
I apologize for my delay in getting back to you.

The idea is nice, the data could be interesting, but the methodology is poor.
It is impossible to replicate your study, therefore I do not believe that this should be published as original research. I would advise rewriting as a research letter as this paper can at best be hypothesis generating.
Full revision is available in the attached file: "Review bmjopen-2023-079681 (PP).pdf "

Best regards
Paolo
Authors' reply:
Dear Paolo,
Thank you for your detailed revision. We truly appreciate your thorough feedback and the time you invested in reviewing our manuscript.
We appreciate your feedback on the methodology. The action research methodology we employed is a well-established approach designed to generate knowledge to solve real-world problems within their specific contexts, involving stakeholders through iterative cycles of implementation and evaluation. We used a mixed methods design that combines quantitative and qualitative data collection, underpinned by a pragmatic approach. Hence, the quality of the study results cannot be judged by the positivist approach, and the results does not need to be replicable.1 Instead, transparency of reporting the methods, and authenticity of the results serves as the quality benchmark against which the validity and reliability of action research are measured.2
We understand your suggestion to consider a Research Letter format. However, after reviewing the journal's options, we could not find this format available. Additionally, we believe presenting our manuscript as a Research Letter is not adequately convey the detailed results of our mixed methods action research.
Again, thank you for taking the time to review our manuscript and providing valuable feedback. We appreciate your constructive comments and have carefully considered your concerns. Please, find our response to the full-version review below:
Reviewers' comments (full revision)
Abstract:
Title: Title is interesting, design of the study is not clear enough
Authors' reply: Revised as suggested by the handling editor.
Abstract:
Objectives: accessible → accessibility
Authors' reply: Revised as suggested.
You can summarize the objectives in customization and effectiveness.
Authors' reply: Thank you for the suggestion. We have revised the objective. However, identifying the learning enhancing-design elements is the core objective of the study.

Design:
Institution-based is too vague. Please summarize at your best the research design.
Authors' reply: Revised as suggested.

Setting:
As per journal guidelines: Generalise; don't use the name of a specific centre but give geographical location if important.
Authors' reply: Revised as suggested.

Interventions:
There are no interventions made in this study, you already explained that you want to develop and evaluate SBES, no need to add the modules. This part can be omitted.
Authors' reply: Revised as suggested.

FULL PAPER
Background:

1.“These include … development opportunities” too long, hard to go through.
2. The introduction is long, 5 paragraph. Make it shorter and more focused on why SBES is important in the humanitarian education setting. The paragraph about the aim is well written.
3. Remove CRIMEDIM long title and put it in the setting.
Authors’ reply: Thank you for your suggestion. The introduction has been edited as you have suggested to eliminate redundancy and improve clarity and maintain focus of the research problem.

Methods:
1. I have to admit, I never heard of this research design, but I have analysed the reference 32 and 33, I believe that there are not enough information to conclude that the method used is a modified version, known as rapid prototyping. Rapid prototyping is described different from ADDIE.
To clarify, the Rapid Prototyping – modified ADDIE (Analysis, Design, Development, Implementation, Evaluation) is a framework for training development, not the study design itself. We apologize for any confusion this may have caused in the original manuscript. In our study, we utilized the ADDIE framework as a basis for our training program development, but the actual study design employed was a mixed methods action research. We now explicitly have stated the study design and framework of the study in the methods section.
2. “The study design consist of … strand”, this sentence is vague. A study design must be clear and easy to be repeated.
Authors’ reply: We appreciate your observation, and we revised the sentence to provide a clearer and more detailed description of the study design.
3. “Participants were purposively sampled”.
Authors’ reply: Thank you for your comment. We have revised the SBES target population and study participants section to include the relevant following details.
a. Please describe how were they sampled.
Authors’ reply: We targeted the training stakeholder by purposive sampling. The participants were contacted through emails initially, given that it was a COVID-19 period. Further communication through the action cycles took place either online or face-to-face based on their availability.
b. Which were the inclusion criteria?
Authors’ reply: Qualitative component: Expert and coordinator of training and education programs in disaster medicine, humanitarian health, and public health training, and e-learning, and instructional design experts. All students from DisasterSISM, The Training Disaster Medicine Trainers (TdmT), and The European Master of Science in Disaster Medicine (EMDM), and Master of Public Health were eligible to participate in the study, as they represent the target audience of the SBES.
Quantitative component: We targeted all the student from TdmT, 2022 to represent the undergraduate and all student from EMDM 2022 to represent post-graduate trainee.
c. How did you reach them?
Authors’ reply: The participants were contacted through emails initially, given that it was a COVID-19 period. Further communication through the action cycles took place either online or face-to-face based on their availability.
d. Which was the response rate?
Authors’ reply: For the quantitative study response rate was 61.4%
4. No need to put the footer with the website of the various software used.
Authors’ reply: The footer has been deleted.
5. “When saturation was achieved”, how do you define saturation?
Authors’ reply: In qualitative research, saturation occurs when no new issues or insights arise, and data repetition suggests that further collection is unnecessary, indicating an adequate sample size. It's the point where gathering more data on a theoretical construct reveals no new properties. 3
6. Which software was used for the statistical analysis?

Authors' reply: Add in the text SPSS version 29 was used "The tests were performed with IBM SPSS version 29 (IBM Corp.;Armonk, New York USA)."
7. SBES Development chapter is structured in a way that follow the ADDIE scheme, why the last point is Action and not Evaluation?
Authors' reply: The rapid prototyping is using the ADDIE scheme in an iterative, non-consecutive cycles and overlapping cycles, with implementation of continuous action for improvement. Hence, we provided the description of the training development, including the action that implemented to improve each step for training development, then we have presented the mixed method evaluation of training.
8. Questionnaire should have been listed as supplementary material.
Authors' reply: Added to the supplementary
9. You should have put here the (CRe-DEPTH) Checklist.
Authors' reply: Thanks for pointing out. We added a statement referring to the checklist at the beginning of the methods section.

Results:
1. Reference should not be present here as you should be just writing the results of your study.
Authors' reply: The reference now has been deleted.
2. "However, the formative evaluation ... humanitarian crises" this seems to me more like a discussion point, here you should be listing the results of the formative evaluation.
Thank you for your suggestions. We have revised this section.
3.SBES effective design validation: this part should be listed as method, together with figure 1
Authors' reply: We apologize for any confusion regarding our use of the term "validation." In our manuscript, we used "validation" to refer to identifying the design elements that facilitate effective learning transfer using SBES. To avoid further confusion, we have removed the term "validation" from the manuscript.
The framework we present in the results section is derived from our qualitative data analysis and can be applied by global educators to develop and evaluate SBES for global public health education and related fields. We believe this framework is best situated in the results section to convey its development and application based on the collected data.
4. From "Figure 1: SBES effective design" to "functionality" is a list of comments from the study participants. Feedback and satisfaction rate should be based on appropriate CRF and surveys.
Authors' reply: The results presented here are derived from the qualitative data collected in the study, which was essential to develop the SBES effective design elements framework.
We also understand the importance of tools used to collect this data. We used several methods and tools to collect the qualitative data, which has been described in the methods section.
In addition, we added the questionnaire that we used to collect the data during summative evaluation in the supplementary document II.
5. "Many students valued the interactivity of SBES", show me how and where, I need to see this in the results.
Authors' reply: Thank you for your comments. These are the results of the qualitative part of the study, in which we have used different methods for data collection. We provided two quotes from two students. We deleted "many" to avoid the confusion in the quantitative results section. In the quantitative results, you can find the interactivity was rated 5.9 in a scale of 7.
6. SBES Effectiveness: how you recruited the students should be in the methodology.
Authors' reply: Thank you for the suggestion. We have moved that to the methods section.
7. "fifty-seven": In general, words should be used for numbers from zero through nine, and numerals should be used from 10 onwards.
Authors' reply: Thank you for the suggestion. We have corrected it in the text.

Discussion:
1. “The SBES evaluation showed high satisfaction rate”, how did you measure it?
Authors’ reply: The satisfaction was one of the questions in the survey of the overall experience. Detailed description of the methods can be found under the subtitle: quantitative data collection. The results presented under the subtitle Reaction in the Quantitative results subsection. See also the details in the supplementary figure.
2. You talk about limitations, but you didn’t mention them in the chapter “Strengths and limitations of this study”.
Authors’ reply: Since the “Strength and limitation chapter” aims to provide bullet points about the methods with limited word count, we have summarized all the limitations of our study in the last point, which were the subjective nature of action research, the limited scope of evaluation, and the limited generalizability. However, all the technical details of each point have been explained in the discussion.
3. “Hence, the evaluation was limited to a single-group quasi-experimental design, which has low internal validity but indicates real-world behaviour.” I believe this sentence summarize the reason why this research paper should be a research letter.
Authors’ reply: Thank you for your feedback. I would like to clarify the scope and methodology of our study. Our research employed a mixed-method action research design, primarily focusing on qualitative analysis with a quantitative component.
The quasi-experimental (pre/post-test) design is one of most commonly used design to evaluate the training success in disaster medicine education.4 While we included a pre/post-test design to measure changes in knowledge scores, hence the success of the SBES, the primary objective was to identify the design elements leading to effective learning transfer through qualitative inputs from different stakeholders through iterative cycles of developing and refining SBES. The framework identified through this process can be used by global public health educators to develop and evaluate similar trainings.
Hence, the aim of quasi-experimental design was not to establish causality but to triangulate data from multiple sources to provide a holistic understanding of the intervention's effectiveness, including effective design elements to consider when developing SBES.
Given the depth and scope of our study, which integrates multiple data sources for evaluation and iterative design processes, we believe it is best suited as a full research paper rather than a research letter. We hope this clarifies our approach and the rationale for the manuscript's format.

Conclusion:
1. How can you say that you reached a successful design?
Authors’ reply: Thank you for your question. We reached a successful design through an iterative cycle of action and evaluation involving different stakeholders. The qualitative evaluation identified successful design elements as identified by stakeholders, which have been incorporated into the SBES design where they did not already exist. Additionally, the SBES received positive reactions from students, evident by the high scores each element of the SBES design, including the overall experience, has received. Finally, there was a significant improvement in knowledge scores post-test compared to the pre-test.4
2. How can you say that this is a bridge, have you explored other interventions?
Authors’ reply: Thank you for your feedback. I would like to address your concern about the exploration of other interventions. SBES has the potential to address many of the training gaps in the field as explained in the introduction. Therefore, this study aimed to design and evaluate an effective SBES to address some of the training gaps within existing disaster medicine education programs. With the increasing use of SBES in global health education, the primary goal of this study was to identify and understand the design elements that contribute to its effectiveness, rather than comparing

it with other interventions. We chose not to explore other interventions as our primary interest was in the detailed examination and enhancement of SBES, which we believe adds significant value given the current gaps in the literature. We hope this clarifies our research focus and the rationale behind our approach.

References:
1.References Apex should be at the end of a sentence and not in the middle of it.
2. I can't access to the reference number 1, it says "Page not found", please report access date.
3. Reference number 3, please report access date.
4. Check reference number 33
5. Remove link to reference number 51
Authors' reply: Thank you for your observation. We have edited the references as suggested.

Reviewer: 2
Dr. Raissa Silva Souza, Universidade Federal de São João del-Rei
Comments to the Author:
Article brings up a topic relevant to continuous education in the healthcare field. The text thoroughly presents the stages of study development, along with results and discussion that shed light on the addressed issue.
Authors' reply: Thank you for your insightful review of our article. We appreciate your acknowledgment of the relevance of the topic and your positive comments on the clarity of our presentation of the study's development stages, results, and discussion.

References:
1. Evered RD. An Assessment of the Scientific Merits of Action Research Author (s): Gerald I . Susman and Roger D . Evered Published by : Sage Publications , Inc . on behalf of the Johnson Graduate School of Management , Stable URL : http://www.jstor.org/stable/23925. Adm Sci Q. 1987;23(4):582–603.
2. Zuber-Skerrit O, Fletcher M. The quality of an action research thesis in the social sciences. Qual Assur Educ. 2007;15(4):413–36.
3. Hennink M, Kaiser BN. Sample sizes for saturation in qualitative research: A systematic review of empirical tests. Soc Sci Med [Internet]. 2022;292:114523. Available from: https://doi.org/10.1016/j.socscimed.2021.114523
4. Stratton SJ. Quasi-Experimental Design (Pre-Test and Post-Test Studies) in Prehospital and Disaster Research. Prehosp Disaster Med. 2019;34(6):573–4.
5. Bakker A, Cai J, English L, Kaiser G, Mesa V, Van Dooren W. Beyond small, medium, or large: points of consideration when interpreting effect sizes. Educ Stud Math. 2019;102(1):1–8.

VERSION 2 – REVIEW

**REVIEWER**	Pallavicini, Paolo<br>Vita-Salute San Raffaele University, School of Medicine
**REVIEW RETURNED**	04-Jun-2024

**GENERAL COMMENTS**	Dear Awsan,<br><br>It is my pleasure to say that I am extremely satisfied of your review.

	Thank you for your extremely precise review, you gave an answer to all of my concerns.<br><br>Best regards<br>P

VERSION 2 – AUTHOR RESPONSE
